# Evaluating Practitioner Training to Improve Competencies and Organizational Practices for Engaging Fathers in Parenting Interventions

**DOI:** 10.1007/s10578-018-0836-2

**Published:** 2018-08-04

**Authors:** M. Burn, L. A. Tully, Y. Jiang, P. J. Piotrowska, D. A. J. Collins, K. Sargeant, D. Hawes, C. Moul, R. K. Lenroot, P. J. Frick, V. Anderson, E. R. Kimonis, M. R. Dadds

**Affiliations:** 1 School of Psychology, University of Sydney, Camperdown, NSW 2006 Australia; 2 School of Psychiatry, Faculty of Medicine, University of New South Wales, Sydney, NSW 2052 Australia; 3 Department of Psychology, Louisiana State University, 236 Audubon Hall, Baton Rouge, LA 70803 USA; 4 Learning Sciences Institute of Australia, Australian Catholic University, Brisbane, QLD 4000 Australia; 5 Royal Children’s Hospital, Murdoch Children’s Research Institute, Parkville, VIC 3052 Australia; 6 Department of Psychology, University of Melbourne, Parkville Campus, Melbourne, VIC 3010 Australia; 7 Department of Paediatrics, University of Melbourne, Parkville Campus, Melbourne, VIC 3010 Australia; 8 School of Psychology, University of New South Wales, Sydney, NSW 2052 Australia

**Keywords:** Parenting programs, Father engagement, Practitioner training, Practitioner competencies

## Abstract

Fathers are consistently underrepresented in parenting interventions and practitioners are an important target for change in interventions to enhance father engagement. This research examined the effects of two practitioner training programs in improving practitioner rated competencies and organizational father-inclusive practices. Two studies were conducted, each with a single group, repeated measures (pre, post and 2-month follow-up) design. Study 1 (*N* = 233) examined the outcomes of face-to-face training in improving practitioner ratings of competencies in engaging fathers, perceived effectiveness and use of father engagement strategies, organizational practices and rates of father engagement. Study 2 (*N* = 356) examined online training using the same outcome measures. Practitioners in both training formats improved in their competencies, organizational practices and rates of father engagement over time, yet those in the online format deteriorated in three competencies from post-training to follow-up. The implications for delivering practitioner training programs to enhance competencies and rates of father engagement are discussed.

## Introduction

Fathers play a vital role in the development and wellbeing of children. There is now significant evidence demonstrating the unique contribution of fathers to child outcomes, both positive and negative [1]. Parenting interventions, which aim to improve the quality and consistency of parenting, are well established as effective for improving child outcomes. However, fathers are consistently underrepresented in parenting interventions [2], despite research demonstrating that father involvement (along with mothers) improves outcomes for parenting and child externalizing behaviors [3]. While there are many reasons for the low rates of father participation—often related to broad socio-ecological issues impacting fatherhood—research has also identified certain practitioner and organizational factors that may play a role, including low levels of practitioner competencies in engaging fathers and low rates of organizational support for father-inclusive practice [4]. In addition, few practitioners report having participated in training programs focused on enhancing skills for engaging fathers [4], yet training is associated with improved practitioner competencies [4–6], and has been found to be a promising approach for increasing rates of father engagement. However, many practitioners find it difficult to access such programs, so online formats are needed in addition to face-to-face formats, to increase the availability of training. As practitioners are an important target for change in interventions to increase father engagement, this research examines the effects of a face-to-face and online training program in enhancing practitioner competencies and organizational practices for engaging fathers.

The majority of studies on parenting interventions do not report rates of father participation [2] and when rates are reported, they are generally low. For example, a review of 28 studies on parenting interventions found only 20% of parents enrolled in parenting interventions were fathers [7]. Practitioner competencies, which refer to a broad combination of knowledge, values, attitudes and skills [8], are likely to play a critical role in influencing rates of father engagement. Although practitioners believe that competencies are important to father engagement, and are very amenable to change [9], one survey found only one quarter of practitioners delivering parenting interventions had high levels of competencies [4]. Organizational factors can also act as important barriers and facilitators for father engagement. These include factors such as level of organizational support for father inclusion [4, 10, 11], and practices such as emphasizing the importance of fathers at intake [4] and offering sessions outside normal working hours [12]. In fact, organizational support for father-inclusive practice (as rated by practitioners) has been found to significantly predict higher rates of father engagement [4].

Specific training in father engagement appears to be important for promoting practitioner competence. To date, three studies have evaluated the outcomes of training programs focused on enhancing practitioner competencies in engaging fathers. Firstly, Scourfield et al. [6] evaluated a 2 day training for social workers delivering statutory child welfare services in the UK. There were significant changes in practitioner-reported confidence in engaging with fathers, from pre-training to 2 month follow-up. In addition, at follow-up there were significant practitioner-reported increases in rates of father engagement across 3 out of 6 measures (for fathers who were not perceived as putting their child at risk). In a second study, Scourfield, Smail, and Butler [13] evaluated a briefer 1 day training to improve practitioner confidence in father engagement, within a single child welfare county in the UK. This evaluation found significant improvements from pre-training to 2 month follow-up in confidence across 12 out of 17 items. Finally, Humphries and Nolan [5] evaluated a 1 day training program for health visitors delivering parenting and infant care services for parents with children 0–5 years. The evaluation showed significant improvements from pre- to post-training in participant knowledge about fathers, attitudes towards fathers and intentions to engage fathers. While improvements were only maintained at 2 month follow-up for attitudes (there was a significant deterioration for knowledge and engagement behavior), follow-up scores were significantly higher than pre-training scores.

Together, the outcomes of these training evaluations provide initial evidence that training may improve practitioner competencies in engaging fathers, with some limited evidence that it may also improve rates of father attendance. In addition to the scarcity of studies and their methodological problems (such as small sample sizes), there are three main limitations of the research to date. Firstly, studies have focused on specific groups of practitioners (i.e., statutory child protection workers and health visitors) and there are no evaluations of training programs which include practitioners from a range of professional disciplines. This is important as parenting interventions are delivered by practitioners with a wide variety of professional backgrounds [4, 14]. Relatedly, by focusing predominantly on the child welfare or home visiting context, training has not yet been evaluated for practitioners who deliver parenting interventions in other, non-statutory settings, such as child and adolescent mental health services and private psychological services. Secondly, no research has examined whether training of practitioners improves organizational support for father-inclusive practice or enhances service-level use of father engagement strategies, despite evidence that organizational factors appear to be important for father engagement [4]. Finally, some of the training programs implemented to date have been lengthy, at up to 2 days in duration, and given demands on practitioners’ time, there is likely to be reduced uptake of lengthy training [15]. Thus, we aimed to examine the outcomes of a brief half-day training program targeting a broad range of professionals delivering parenting interventions, and to examine changes in practitioner competencies and organizational support for father-inclusive practice.

Practitioners may be more likely to participate in a brief, rather than intensive, training program; however there remain many barriers to accessing face-to-face training [16]. In addition, it is difficult to disseminate face-to-face training widely, given the intensive time and resources involved in delivering such programs. Providing training via the internet may enhance reach, as it is convenient and cost-effective [17, 18], and there is emerging evidence that online training can have similar effects to face-to-face delivery for psychologists and other health professionals [19, 20]. Online training therefore has the potential for widespread dissemination, and is a promising method for improving practitioner competencies in engaging fathers in the community. Thus, the current research also aimed to develop and evaluate an online format of the father engagement training.

The aim of this research was to examine the outcomes of a face-to-face (Study 1) and an online training program (Study 2) for practitioners who deliver parenting interventions, as measured by changes in practitioner-reported competencies and organizational practices for engaging fathers. The training program was part of a larger project called *Like Father Like Son*, which aimed to enhance the engagement of fathers in evidence-based parenting interventions in Australia. While it would have been ideal to compare the outcomes of the two training formats within the one study, due to differences in study samples and methodology for the different formats, it was not possible to contrast them directly and instead they were examined in two separate studies. For both studies, it was expected that there would be significant improvements in self-reported: (1) confidence in working with fathers, competence in use of father engagement strategies, and perceived effectiveness of strategies from pre- to post-training assessment, and these improvements would be maintained at the 2 month follow-up (Hypothesis 1); (2) frequency of using father engagement strategies from pre-training to 2 month follow-up (Hypothesis 2); (3) organizational use of strategies to enhance father engagement and organizational support for father-inclusive practice from pre-training to 2 month follow-up (Hypothesis 3) and; (4) rates of practitioner-reported father engagement from pre-training to 2 month follow-up (Hypothesis 4). The design for both studies was a single group, repeated measures, non-randomized trial, with assessments at pre-, post- and 2 month follow-up. Reporting of these studies is in accordance with the TREND statement for the reporting of intervention evaluation studies with non-randomized designs [21].

## Study 1

### Method

#### Participants

**Fig. 1:**
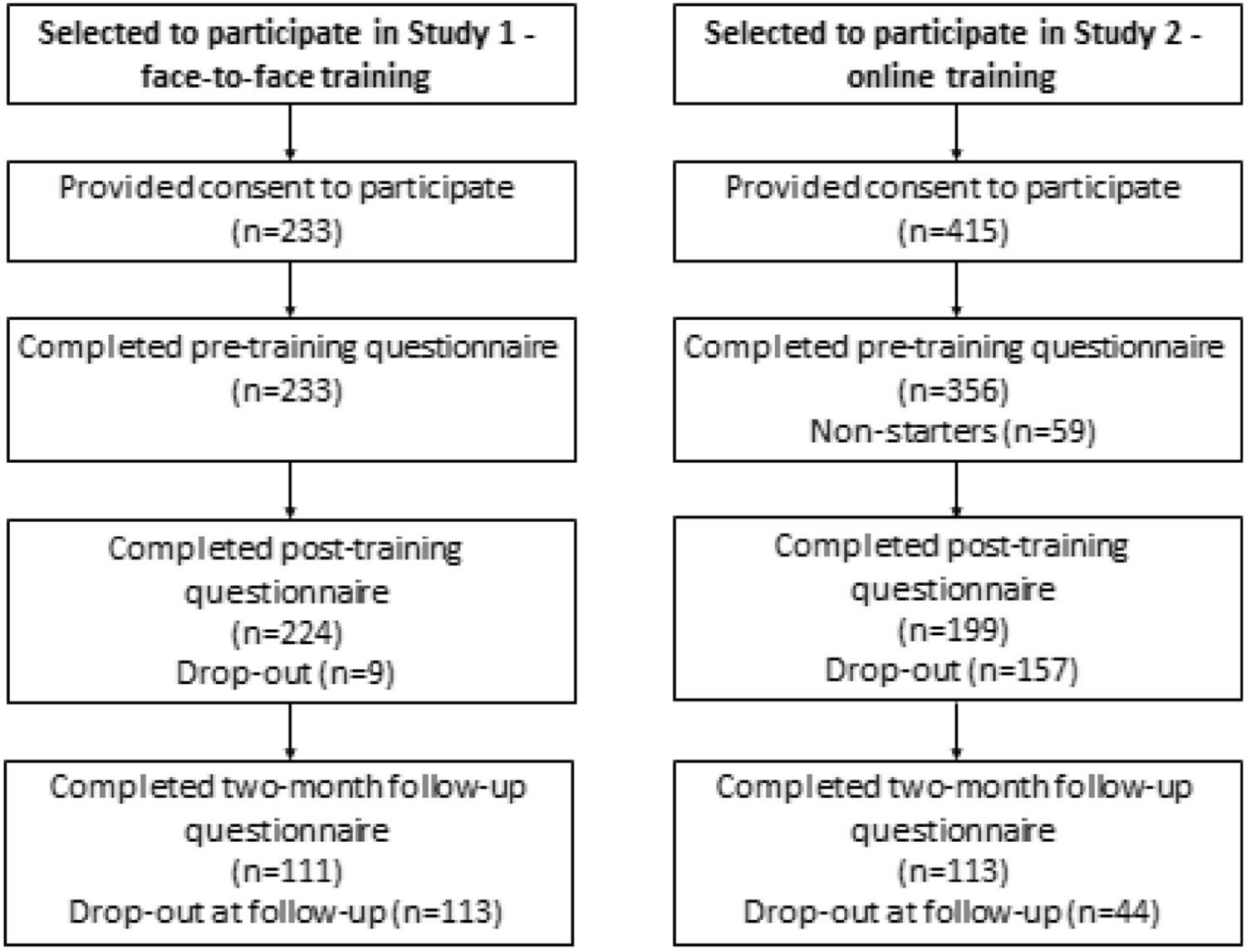
Flow chart for recruitment into the face-to-face training and online training

Participants were eligible for participation in the face-to-face training (FFT) if they worked for an organization in Australia that delivered parenting interventions. Practitioners who conducted clinical work with families were the main target of the intervention, however, support staff such as managers and administrative staff were also eligible to participate to enhance the engagement of fathers at the organizational level. For example, administrative staff who speak with families on the phone to arrange appointments may have the opportunity to increase father engagement by encouraging both fathers and mothers to attend sessions. In total, 223 participants gave consent to participate in the FFT and completed the pre-training assessment. Figure 1 displays the flow chart for recruitment to the study. The demographic and professional characteristics of respondents are reported in Table 1. Just over one-quarter were psychologists, one-fifth were social workers and the remainder were counsellors, caseworkers, family support workers, nurses, managers, educators and ‘other’ professions (these included psychiatrists, general practitioners, occupational therapists, speech therapists). The broad range of professional groups in the sample is indicative of the diverse practitioners who deliver parenting interventions in Australia. Half of the sample worked for a non-government organization. The majority of participants were female (84.5%) and worked in a direct service provision role with families (82.7%). Participants had on average 9.1 years (range 0–39) of experience working with families, and around 1 in 5 had previously participated in father engagement training.

**Table 1:** Sample characteristics for face-to-face and online training groups

Variable	Study 1 FFT (*n* = 233)	Study 2 OT (*n* = 356)
	*M* (SD)	*M* (SD)
Age	39.2 (11.4)	38.2 (11.2)
Years of experience	9.1 (7.7)	8.8 (8.2)

Variable	Study 1 FFT (*n* = 233)	Study 2 OT (*n* = 356)
	*n* (%)	*n* (%)
Female	197 (84.5)	305 (85.7)
Direct work with families	191 (82.7)	318 (89.3)
Previous training in engaging fathers	45 (19.2)	67 (18.8)
Profession		
Psychologist	63 (27.3)	167 (46.9)
Social worker	49 (21.2)	49 (13.8)
Counsellor	18 (7.8)	14 (3.9)
Caseworker	18 (7.8)	20 (5.6)
Family support worker	18 (7.8)	38 (10.7)
Nurse	6 (2.6)	20 (5.6)
Manager	4 (1.7)	9 (2.5)
Educator	12 (5.2)	8 (2.2)
Mediator	4 (1.7)	7 (2.0)
Other	39 (16.9)	24 (6.7)
Organization type		
Child/family mental health	47 (20.2)	46 (12.9)
Other government	29 (12.4)	68 (19.1)
Non-government organization	119 (51.1)	133 (37.4)
University	18 (7.7)	10 (2.8)
Private practice	10 (4.3)	79 (22.2)
Other	10 (4.3)	20 (5.6)
Location of workplace^a^		
New South Wales	123 (52.6)	173 (53.2)
Queensland	20 (8.5)	65 (20.0)
South Australia	14 (6.0)	14 (4.3)
Victoria	10 (4.3)	55 (16.9)
Western Australia	8 (3.4)	7 (2.2)
Northern Territory	17 (7.3)	1 (0.3)
Australian Capital Territory	19 (8.1)	6 (1.8)
Tasmania	23 (9.8)	4 (1.2)

^a^Location based on postcode. For OT *n* = 325 as some practitioners did not know postcode for workplace

*FFT* face-to-face training, *OT* online training

#### Measures

Practitioners completed questions about their sociodemographic (i.e., age, gender) and professional characteristics (years of experience, profession, current organization, previous participation in training on father engagement). Practitioners also completed the Father Engagement Questionnaire (FEQ) [22]. The FEQ has 31 items, across five scales with acceptable test–retest reliabilities and internal consistencies [22]. The scales are described below.

*Confidence in Engaging Fathers* scale (‘Confidence’ scale) included 9 items measuring practitioner confidence in various aspects of father engagement. Example items include engaging fathers who are reluctant to attend, dealing with resistance from fathers, managing distress from fathers, communicating with fathers, and managing conflict between mothers and fathers. Participants rated each item on a five-point scale from *not at all confident* (1) to *extremely confident* (5). For this and all other scales in the FEQ, participants who had never worked clinically with families (e.g. support staff) could select the option *not applicable*/*never worked directly with families*. This scale was administered at pre, post and at 2 month follow-up.

*Competence in Using Father Engagement Strategies* scale (‘Competence’ scale) included 5 items measuring competence in implementing father engagement strategies. Example items include directly inviting fathers who are reluctant to attend, listening to fathers and exploring their barriers to engagement, and managing conflict (practitioner-client and mother-father). Participants rated their perceived competence using a five-point rating scale ranging from *not at all competent* (1) to *extremely competent* (5). This scale was administered at pre, post and at 2 month follow-up.

*Perceived Effectiveness of Father Engagement Strategies* scale (‘Perceived effectiveness’ scale) included 5 items measuring perceived effectiveness of the same father engagement strategies as those in the Competence scale. Participants were asked to rate their perceived effectiveness of the strategies to engage fathers using a five-point rating scale ranging from *not at all effective* (1) to *extremely effective* (5). This scale was administered at pre, post and at 2 month follow-up.

*Frequency of Use of Father Engagement Strategies* scale (‘Frequency of use’ scale) included 5 items measuring frequency of use of the same father engagement strategies as those in the Competence and Perceived Effectiveness scales. Participants were asked to rate their frequency of use using a five-point rating scale ranging from *never* (1) to *always* (5). This scale was administered at pre and 2 month follow-up.

*Organizational Practices for Father Engagement* scale (‘Organizational practices’ scale) included 4 items and measured practitioner-reported organizational use of father engagement strategies. Example items included: emphasizing the importance of father attendance at intake and offering sessions outside work hours to enable fathers to attend. Participants were asked to rate their service or program’s use of father engagement strategies over the past 2 months using a five-point rating scale ranging from *never* (1) to *always* (5). This scale was administered at pre and 2 month follow-up.

Practitioners were asked at pre-training and two-month follow-up about the frequency of father attendance at their service/program. They were asked to choose the statement that best described their work with fathers over the last 2 months, using the scale *fathers never attend* (1), *rarely attend* (2), *sometimes attend* (3), *often attend* (4), and *always attend* (5). Practitioners were asked at pre-training and 2 month follow-up about their perceptions of organizational support for father-inclusive practice. They were asked to rate the level of support they received from their organization for engaging fathers using a five-point scale from *not at all supportive* (1) to *extremely supportive* (5). Finally, at post-training only, participants were asked: *Overall, how helpful was the training to your work with fathers?* They provided responses on a five-point rating scale ranging from *not at all helpful* (1) to *extremely helpful* (5).

#### Procedures

The Human Research Ethics Committee (HREC) at the University of Sydney provided ethical approval for this study. As some FFT programs were run within child and family services around Australia, local ethics committee approvals were also obtained where necessary. Organizations were recruited via two methods. Firstly, 12 child and family organizations were contacted directly via email and invited to hold a FFT at their service. Of those organizations contacted, six agreed and held a FFT in their service. Secondly, flyers and advertisements directed practitioners to a project website which included information about the FFT. Advertisements were distributed via social media and professional organizations (such as the Australian Psychological Society and the Australian Association of Social Workers). Nine FFT programs were arranged through practitioners seeing advertising information and then contacting the researchers.

During a 13 month period (from August 2016 to September 2017), 15 FFT programs were conducted free of charge to organizations and participants across all eight Australian states/territories: Australian Capital Territory (*n* = 1), New South Wales (*n* = 8), Western Australia (*n* = 1), Queensland (*n* = 1), Victoria (*n* = 1), Tasmania (*n* = 1), South Australia (*n* = 1) and Northern Territory (*n* = 1). Seven of the FFTs were conducted with staff from a single organization (five of which had a manager present at the training), and the remaining eight FFTs had staff from several organizations in attendance. Groups ranged in size from 8 to 24 participants. At the time of the FFT, participants read an information sheet and signed a consent form and then completed pre-training questionnaires. After the training, participants completed the post-training questionnaires and provided their email addresses which were used to send training certificates and a link for the online follow-up questionnaires, which were completed 2 months after training, using Qualtrics™ online survey software. To ensure participant data remained anonymous, email addresses were not linked to questionnaire data. One email reminder was sent to non-respondents, one week after the initial email was sent. The average length of time from training to completion of two-month follow-up questionnaires was 67.5 days (SD = 11.1, range 55–123).

#### Content of the FFT Program

The FFT was developed by psychologists and child and family practitioners who were experienced in working clinically with families and fathers. The training was developed based on social cognitive theory (SCT), according to which there is reciprocal determinism between behavior, cognitive/other personal factors, and the environment, which interact to influence each other [23]. The training aimed to increase participants’ self-efficacy to engage fathers, as well as their ability to self-evaluate their own learning regarding father engagement skills, in order to modify their skill implementation (self-regulation), within their individual work context. In keeping with SCT, the FFT was developed to be an interactive skills training workshop involving modelling, rehearsal and feedback on skills. The specific father engagement strategies included in the training program had been used to train clinical psychology students working in a University-based clinic that delivered parenting interventions for child behavioral problems over a period of several years, and this service was highly successful in engaging fathers. It achieved an average rate of father engagement of 72% (representing percentage of total sessions attended by fathers), which was significantly higher than the average rate of father engagement of 48% across ten child and family mental health services in Australia [24]. A review of the literature on father engagement was also undertaken to inform the development of the content of the program.

The FFT was 4.5 h in duration and included a combination of didactic presentations, use of video vignettes (including actors in the role of parents and practitioners demonstrating key skills), active skills training, and group activities and discussion on five topics: understanding research on father engagement; exploring barriers to father engagement; developing positive engagement strategies; building confidence in managing conflict; and, planning for future father-inclusive practice. Participants had access to a workbook and completed workbook activities throughout the training. See Table 2 for a description of the content and activities included in the training.

**Table 2:** Content included in the face-to-face and online training

Section	Learning objective and content	Activities in FFT	Activities in OT
1. Research background	Learning objective: increased understanding of the evidence-based rationale for engaging fathers in parenting programs Information presented on: • Emerging research regarding fathers and their impact on child outcomes • Research regarding father preferences for parenting interventions and perceptions of barriers to engagement • Research regarding practitioners’ competencies	Large group activities which involve guessing statistics from research about father engagement. This is followed by group discussion about practice implications	No activities
2. Barriers to father engagement	Learning objective: increased knowledge about the potential barriers to father engagement Interactive vignettes and questions focused on: • Exploring possible barriers to father engagement • Reflecting on the impact of barriers to engagement within the participant’s own professional context	Participants watch video vignettes and are asked to imagine what the father and practitioner are thinking and feeling. Large group discussion about father and practitioner factors that may be barriers to father participation. Large group discussion about additional barriers to father engagement	Participants watch video vignettes and complete workbook activities to identify father and practitioner barriers to father engagement, Further workbook activity to identify additional barriers to father engagement
3. Positive engagement strategies	Learning objective: increased knowledge and skills about how to invite fathers to participate in parenting programs and keep them engaged Information presented on • Strategies for engaging fathers through mothers, including 1. Identifying who is in the core parenting team 2. Helping mothers to identify barriers to father engagement 3. Emphasising the importance of father participation 4. Empowering the mother to make decisions • Building skills for directly inviting fathers to attend parenting interventions, including 1. Explaining the service/intervention 2. Emphasising the importance of the father 3. Talking to his story through active listening (rather than problem solving) 4. Being gutsy (confident and direct about the need to include him • Building skills for positive engagement with both parents, including 1. Giving each parent an equal opportunity to speak 2. Taking a stance of curiosity and empathy 3. Staying with each parents until you have a complete story 4. Reflecting to check for clarification 5. Summarising a shared understanding • Building skills for establishing a ‘team approach’ to working with both parents	Participants watch video vignettes of a practitioner engaging the father through the mother. Participants complete an individual activity identifying ways to talk with mothers about father engagement Roleplay involving entire group where participants practice directly inviting fathers to participate in a parenting intervention, using key steps covered. This is followed by feedback and group discussion One participant plays the practitioner in a group roleplay to demonstrate challenges that practitioners may face in engaging both parents (especially when one parent is disengaged). This is followed by group discussion and then the facilitator models key steps for active engagement of both parents	Participants watch video vignettes of a practitioner engaging the father through the mother. Participants complete a workbook activity identifying ways to talk with mothers about father engagement Participants watch video vignettes of a practitioner directly inviting fathers to participate in a parenting intervention. Participants complete a workbook activity to outline how they could use the strategies to engage both parents Participants watch video vignettes of a practitioner experiencing difficulties in using positive engagement strategies, and a second showing success with these strategies. Participants complete an activity to outline how they could use the strategies to engage both parents
4. Building confidence in managing conflict	Learning Objective: Increased skills and confidence in managing conflict when working with parents Information presented on • Identifying when conflict is emerging • Exploring feelings underlying anger, hostility and blame • Formulating procedures for managing escalating conflict (parent-practitioner or parent-parent) • Exploring the purpose of modelling repair after conflict	Participants watch video vignettes of conflict scenarios. Large group discussion about conflict and its impact on practice, and feelings underlying anger, hostility and blame Following a demonstration, participants roleplay (in small groups) strategies for managing escalating conflict, followed by feedback Large group discussion about the importance of modelling repair	Participants watch video vignettes of a practitioner managing conflict in a session. Participants complete a workbook activity on conflict and its impact on practice Participants complete a workbook activity on potential emotions underlying anger, hostility and blame Participants reflect on their past practice and evaluate how they dealt with conflict and how they could do this differently Participants complete a workbook activity to outline how they could use the strategies to manage conflict in a session
5. Planning for future father inclusive practice	Learning Objective: Increased awareness of how to promote and maintain ongoing father-inclusive practice in your own workplace, team, and organization/service Information presented on strategies for increasing father-inclusive practice • At an individual level • At a team level • At an organizational level	Small group activities to brainstorm strategies for increasing father-inclusive practice at the individual level, team level and organizational level. This is followed by large group discussion about strategies	Workbook activity to brainstorm strategies to increase father-inclusive practice at individual, team and organizational level

*FFT* face-to-face training, *OT* online training

Two staff (one male and one female) facilitated each training workshop. Facilitators were experienced psychologists and child and family workers, and they followed a facilitator’s manual to deliver the training. Based on a facilitator-rated protocol adherence checklist, there was on average 93.8% adherence to manual content, across all training workshops and facilitators, representing a high overall level of practitioner adherence to manual content.

Prior to commencing recruitment, the FFT was pilot tested to ensure clarity of content, participants’ perceptions of the activities and roleplays. The pilot testing involved 23 participants in two groups and feedback was obtained via a survey and a brief focus group session at the completion of the training. On the basis of the feedback from the pilot tests, minor changes were made to the FFT including modifying the roleplays to ensure clarity of the learning objectives, along with changes to the workbook.

#### Statistical Analysis

To examine whether attrition from pre- to post-training and post-training to follow-up was likely to have influenced results, participants who dropped out were compared to those who remained on their baseline characteristics and pre-training scores.

Analyses of short-term training effects (pre- to post-training) were conducted using repeated measures MANOVA with confidence, competence and perceived effectiveness as the dependent variables. These variables were included in a MANOVA as they were conceptually related and moderately correlated. Significant multivariate main effects for time were further examined by exploring the univariate main effects. To examine longer-term training effects on these variables, a repeated measures MANOVA was conducted (across pre-, post-, and follow-up) with significant multivariate main effects examined by univariate ANOVAs, and pairwise comparisons with Bonferroni corrections which compared mean scores on the three dependent variables at pre-training versus follow-up and post-training versus follow-up.

A repeated measures MANOVA was also conducted with frequency of use of engagement strategies and organizational use of engagement strategies as the dependent variables, to examine long-term changes from pre-training to follow-up (these variables were only assessed at these two time points). Significant multivariate main effects for time were further examined by exploring univariate main effects. Effect sizes were reported using SPSS partial eta square with 0.01 considered a small effect, 0.06 a moderate effect and 0.14 a large effect [25]. An a priori sample size calculation was performed using G*Power 3.1 [26]. A sample size of 54 participants was required at follow-up in Study 1 and 2 in order to detect a small effect for repeated measures MANOVAs (power = 0.80, α = 0.05).

A Wilcoxon signed rank test was used to compare practitioners’ reports of father engagement between pre-training and follow-up. A paired t-test was used to compare ratings of organizational support for father-inclusive practice between pre-training and follow-up. Finally, practitioners’ post-training ratings of helpfulness were examined via descriptive statistics.

All analyses were run with and without those training participants who did not work clinically with families, as it was anticipated that their inclusion might impact on the effects of the training (participants were able to select ‘not applicable responses’ to questions in the questionnaire that they felt did not apply to them, although not all participants selected this response). As exclusion of these practitioners did not change the pattern of results, all analyses reported include participants who did not work directly with fathers.

### Results

The attrition rate for the FFT sample from at post-training was 3.9% (9/233) with a further 50.4% dropping out at follow-up (113/224) (see Fig. 1). Given the small number dropping out at post-training, drop out was combined across post- and follow-up, and analysis examined whether the 122 who dropped out at either phase differed from those who completed both phases. No differences in baseline characteristics or pre-training variables were found.

Table 3 presents the means and standard deviations for pre-training, post-training and 2 month follow-up on each of the five outcome measures for the FFT, along with *F* values for univariate repeated measures ANOVA and partial eta squared effect sizes.

**Table 3:** Means and standard deviations for face-to-face and online training groups at pre-training, post-training and 2 month follow-up

	Pre-training	Post-training	2 month follow-up	Short-term training effects		Long-term training effects	
				Univariate *F*	Partial eta squared	Univariate *F*	Partial eta squared
Study 1—FFT							
Confidence	3.01 (0.56)	3.67 (0.45)	3.67 (0.51)	366.05***	0.64	136.70***	0.57
Competence	3.29 (0.60)	3.84 (0.51)	3.92 (0.59)	163.64***	0.44	77.10***	0.40
Perceived effectiveness	3.81(0.58)	4.32 (0.53)	4.29 (0.57)	140.38***	0.41	60.72***	0.37
Practitioner strategy use	3.06 (0.86)	N/A	3.52 (1.01)	–	–	31.68***	0.25
Service use of strategies	3.07 (0.85)	N/A	3.54 (0.82)	–	–	30.52***	0.25
Study 2—OT							
Confidence	2.99 (0.59)	3.83 (0.56)	3.54 (0.55)	593.51***	0.75	220.51***	0.67
Competence	3.36 (0.65)	4.03 (0.64)	3.82 (0.59)	273.28***	0.58	89.62***	0.45
Perceived effectiveness	3.89 (0.62)	4.52 (0.51)	4.17 (0.51)	215.26***	0.53	99.14***	0.47
Practitioner strategy use	2.98 (0.93)	N/A	3.57 (0.92)	–	–	46.24***	0.31
Service use of strategies	3.08 (0.95)	N/A	3.54 (0.93)	–	–	28.89***	0.22

FFT face-to-face training; OT online training; N/A not assessed at post-training

***p < 0.001

Repeated measures MANOVA (including confidence, competence and perceived effectiveness) of short-term training effects (pre- to post-training) found significant multivariate main effects, *F* (3, 202) = 133.89, *p* < 0.001, with univariate ANOVAs showing means on all dependent variables increased from pre- to post-training. Analysis of long-term training effects (using pre-, post- and follow-up scores on these variables) similarly showed a significant multivariate main effect for time, *F* (6, 100) = 51.33, *p* < 0.001, and univariate ANOVAs showed a significant main effect for time across all three outcomes. Planned comparisons revealed that follow-up scores were significantly higher than pre-training scores for competence (*t* = 0.65, *p* < 0.001), confidence (*t* = 0.67, *p* < 0.001) and perceived effectiveness (*t* = 0.55, *p* < 0.001), and there were no significant differences between post-training and follow-up scores. This indicates that improvements in practitioner ratings on these measures at post-training were maintained at follow-up. Effect sizes ranged from 0.41 to 0.64 in the short-term, and 0.37–0.57 in the longer-term (see Table 3), corresponding to large effects of the FFT on training outcomes over time.

The repeated measures MANOVA for practitioner strategy use and organizational practice with regards to strategy use (assessed at pre-training and follow-up), showed a significant multivariate effect for time, *F* (2, 92) = 25.11, *p* < 0.001, and the univariate main effects for time were significant for both outcomes. Analysis of means revealed significant increases in practitioners’ ratings of their own frequency of use of father engagement strategies as well as organizational father engagement practices from pre-training to follow-up, with large effect sizes (see Table 3). In addition, a paired samples t-test revealed significant improvements from pre-training (*M* = 3.72, SD = 0.89) to follow-up (*M* = 3.89, SD = 0.88) in ratings of organizational support for father-inclusive practice, *t* (118) = − 2.19, *p* = 0.030.

Analysis of practitioner reports of frequency of father attendance rates found that there was a significant change in scores from pre-training (*never* 4.7%, *rarely* 29.0%, *sometimes* 47.7%, *often* 16.6%, *always*, 2.1%) to follow-up (*never* 3.0%, *rarely* 15.0%, *sometimes* 52.0%, *often* 24.0%, *always* 6.0%), with Wilcoxon Signed Rank test *z* = − 4.63, *p* < 0.001. This indicated that the practitioner-reported ratings of father attendance increased from pre-training to follow-up.

FFT participants rated the helpfulness of training an average of 4.1/5 (SD = 0.68) at post-training which corresponded to a rating of ‘very helpful’ on average.

### Discussion

Study 1 examined the outcomes of a brief face-to-face training (FFT) program in changing practitioner competencies and organizational practices. Overall, the FFT was promising in increasing confidence, competence and perceived effectiveness of father engagement strategies from pre- to post-training, and these improvements were maintained at two-month follow-up, providing support for hypothesis 1. The FFT also resulted in improvements in practitioner reports of father engagement strategies and organizational father-engagement practices as well as organizational support for father-inclusive practice from pre-training to follow-up, providing support for hypotheses 2 and 3. Finally, practitioners in the FFT reported improvements in the rate of father engagement from pre- to follow-up, in line with hypothesis 4. The effect sizes for changes in practitioner competencies were in the large range, suggesting that this brief training offers considerable promise in enhancing the father engagement practices of a wide range of practitioners delivering parenting interventions, as well as rate of father engagement. However, face-to-face training will not be accessible for all practitioners (16) and there are also costs for delivery in terms of facilitator training and time, so these factors may limit the dissemination of the program, and points to the need for online training.

## Study 2

### Participants

The same eligibility criteria as for the FFT applied for participants in the online training (OT). In total, 415 participants gave initial consent to participating in the OT and 356 completed pre-training assessment. Figure 1 displays the flow chart for recruitment to the study. The demographic and professional characteristics of respondents are reported in Table 1. Almost half of OT participants were psychologists, 13% were social workers, and the remainder were counsellors, caseworkers, family support workers, nurses, managers, educators, mediators and ‘other’ professions. Just over one-third worked for a non-government organization. The majority of participants (85.7%) were female, and most worked in a direct service provision role with families (89.3%). Participants had on average 8.8 years (range: 0–40) of experience working with families, and just under 1 in 5 reported previous participation in father engagement training.

### Measures

Participants in Study 2 completed the same measures as Study 1.

### Procedures

The University of Sydney HREC provided ethics approval for the study. Participants were recruited into the OT via flyers and advertisements; these directed practitioners to a dedicated project website which included detailed information about the training. Flyers and advertisements were distributed online via social media, websites of professional organizations (e.g., Australian Psychological Society, Australian Association of Social Workers), and sent to a range of child and family organizations around Australia. After being directed to the project website, interested participants read the online participant information statement and indicated their online consent by clicking ‘I agree’. Participants then completed the pre-training questionnaires online (using Qualtrics™ survey software), after which they could immediately participate in the online training. Following training, participants completed the post-training questionnaires and then entered their email to enable the training certificate and the link to the 2 month follow-up questionnaires to be sent. While the study was designed so that post-training questionnaires could be completed immediately after viewing the training video, there was on average 6.9 days (SD = 20.7, range 0–172) between completion of the pre- and post-training questionnaires. Just over half (55.3%) of participants completed pre- and post-training questionnaires on the same day, with the majority (79.4%) completing both questionnaires within 1 week. Only 4.0% took more than 1 month to complete post-training questionnaires. One email reminder was sent to non-responders to prompt completion of post-training questionnaires.

Participants were sent a link to complete follow-up questionnaires online via Qualtrics 2 months after completion of post-training questionnaires. One email reminder was sent to non-responders, one week after the initial email was sent. The average length of time to completion of the follow-up questionnaires was 65.4 days (SD = 6.5, range 37.0–89.0). Enrollment in the OT was open for a 15 month period from August 2016 to November 2017.

### Content of the OT Program

The OT was also based on SCT, and included the same content as the FFT through the provision of audio and visual materials, to create an interactive, engaging training program. The videos included didactic presentations, along with vignettes of practitioners and families that were used to demonstrate key skills. Participants were prompted to download a workbook and pause the video to complete activities throughout the training so they could actively engage with the material and apply it to their own practice. The OT took participants approximately 2 h to complete, which was less than half the time for the FFT, as it did not include active skills training in the form of rehearsal and feedback on roleplay of skills. See Table 2 for a detailed description of the OT content and activities.

Prior to the commencement of the research study, the OT was pilot tested to ensure clarity of video content and workbook activities and ease of completion. The OT was pilot tested with seven participants and feedback was obtained by survey and qualitative interview. On the basis of the feedback from the pilot tests, minor changes were made to the content of the OT videos to ensure clarity of learning objectives.

### Statistical Analysis

All analyses for Study 2 were the same as those described in Study 1.

### Results

The attrition rate for the OT sample from pre- to post-training was 44.1% (157/356) and a further 22.1% (44/199) dropped out at follow-up (see Fig. 1). Examination of drop out at post-training found one significant difference between drop outs and completers across all baseline characteristics and pre-training variables: drop outs were more likely to not work directly with families (15.7%) compared to those who remained (7.0%), χ^2^ (1, *N* = 356) = 6.26, *p* = 0.02. At follow-up, those who dropped out had significantly lower pre-training confidence ratings (*M* = 2.87) than those who remained (*M* = 3.04), *t* (345) = 2.28, *p* = 0.02. There were no other differences across baseline characteristics and pre-training variables.

Table 3 presents the means and standard deviations for pre-training, post-training and two-month follow-up on each of the five outcome measures for the OT, along with *F* values for univariate repeated measures ANOVAs and partial eta squared effect sizes. For the OT sample, repeated measures MANOVA (confidence, competence and perceived effectiveness) of short-term training effects (pre- to post-training) found significant multivariate main effects, *F* (3, 192) = 255.88, *p* < 0.001, with univariate ANOVAs showing mean scores on all dependent variables increased significantly from pre- to post-training. Analysis of long-term training effects (using pre-, post- and follow-up scores) on these variables showed a significant multivariate main effect for time, *F* (6, 105) = 89.89, *p* < .001, and univariate ANOVAs showed a significant main effect for time across all three outcomes. Planned comparisons revealed that scores at follow-up were significantly higher than scores at pre-training for competence (*t* = 0.51, *p* < 0.001), confidence (*t* = 0.66, *p* < 0.001) and perceived effectiveness (*t* = 0.36, *p* < 0.001). However, scores significantly *decreased* from post-training to follow-up on competence (*t* = − 0.13, *p* = 0.03), confidence (*t* = − 0.24, *p* < 0.001) and perceived effectiveness (*t* = − 0.37, *p* < 0.01), indicating a deterioration in improvements from post-training to follow-up. Effect sizes ranged from 0.53 to 0.75 in the short-term and 0.47–0.67 in the longer-term (see Table 3), corresponding to large effects of training in the short- and longer-term.

The repeated measures MANOVA for practitioner strategy use and organizational strategy use (assessed at pre-training and follow-up), showed a multivariate effect for time, *F* (2, 101) = 29.42, *p* < 0.001, and univariate main effects for time were significant for both outcomes. There were significant increases in practitioners’ ratings of their own frequency of use of father engagement strategies as well as organizational practices from pre-training to follow-up, with large effect sizes (see Table 3.). However, a paired samples t-test revealed no significant changes from pre-training (*M* = 3.63, SD = 0.86) to follow-up (*M* = 3.61, SD = 0.78) in ratings of organizational support for father-inclusive practice, *t*(109) = 0.27, *p* = 0.78.

Analysis of practitioner reports of frequency of father attendance found there were significant changes in scores from pre-training (*never* 1.9%, *rarely* 33.0%, *sometimes* 43.3%, *often* 15.7%, *always* 6.1%) to follow-up (*never* 3.8%, *rarely* 17.9%, *sometimes* 46.2%, *often* 28.3%, *always* 3.8%), with Wilcoxon Signed Rank test *z* = − 2.66, *p* = 0.008, indicating improvements in practitioner-reported ratings of father attendance from pre-training to follow-up. OT participants rated the training an average of 4.1/5 (SD = 0.68) at post-training, corresponding to a rating of ‘very helpful’ on average.

### Discussion

Study 2 examined the outcomes of an online training (OT) program in changing practitioner competencies and organizational practices relating to father engagement. The OT was promising in increasing confidence, competence and perceived effectiveness of father engagement strategies from pre- to post-training. Although these improvements were not maintained at follow-up (for the 56.7% of post-training completers who went on to complete 2 month follow-up questionnaires), follow-up scores were nevertheless significantly higher than pre-training scores, indicating an overall improvement in these competencies as a result of the training, and providing partial support for hypothesis 1. The OT was also promising in improving practitioner use of father engagement strategies and organizational father-engagement practices, but not organizational support for father-inclusive practice, from pre-training to follow-up, in support of hypothesis 2 and partial support of hypothesis 3 respectively. Finally, practitioners in the OT reported improved rates of father engagement from pre-training to follow-up, supporting hypothesis 4. The effect sizes for changes in practitioner competencies were large for short-term and longer-term changes. Thus, while some outcomes did not indicate maintenance of training effects in the longer-term, the effect sizes indicate that the training may still have the potential to enhance practitioner competencies and rates of father engagement.

## Overall Discussion

This research examined the outcomes of two brief training programs—face-to-face and online—for enhancing practitioner competencies and organizational practices in relation to father engagement. These were the first studies to evaluate the effects of father engagement training for practitioners delivering parenting interventions across a range of professions and organizational contexts. Overall, there were significant improvements from pre- to post-training, and pre to follow-up, across several practitioner-reported competencies, as well as organizational practices and rate of father engagement. The findings indicate that both formats of the training program were promising and resulted in high levels of practitioner satisfaction, although the less intensive OT did not show maintenance of training effects on three measures of practitioner competencies from post-training to follow-up. Despite this, both programs resulted in significant improvements in practitioners’ ratings of father engagement at follow-up relative to pre-training. These findings support previous research studies which also found significant improvements in practitioner competencies after participation in training [5, 6, 13] as well as significant changes in rates of father engagement [6].

The differences in maintenance of training effects from post-training to follow-up between the two forms of training are likely to be explained by the program characteristics, particularly the level of program intensity and interaction that each allows. Specifically, the deterioration from post-training to follow-up for OT across ratings of confidence, competence and perceived effectiveness, which was not observed in the FFT, may have been due to the shorter training time and/or lack of active skills training in OT. While participants in the OT were able to see demonstrations of effective use of strategies and were asked to think about how these skills could be applied to their own context and to set goals for change, there was no direct opportunity for rehearsal of skills and feedback, which was integral to the FFT. This may have resulted in practitioners experiencing challenges in skill implementation following the OT, leading to reduced confidence, competence and perceived effectiveness in using these strategies (compared to post-training). However, practitioners’ ratings on these competencies at follow-up were still significantly higher than pre-training, suggesting there was a positive effect of training overall. Furthermore, this deterioration did not appear to adversely affect father engagement, as practitioner reported rate of father engagement were significantly higher at follow-up than pre-training.

In order to avoid deterioration effects for online training programs, future programs may need to include additional components to assist in skill application, such as follow-up with a trained facilitator, or receiving feedback on skills demonstration. While such additions may make the training more resource intensive due to the need for expert consultation, there may be methods to minimize this, such as support provided only for those who request assistance or for those who do not demonstrate skill improvement following training. Future research could explore the effectiveness of providing further support for those who require it, through low-cost methods such as email coaching or providing expert feedback on video-recorded skills practice. Research could also examine whether additional support reduces training drop out, since practitioners with lower levels of confidence were more likely to drop out of the OT by follow-up. Notwithstanding the deterioration effect from post-training to follow-up, given that OT was only 2 h in duration, and able to reach a wide array of professionals, the large effect sizes for changes in practitioner competencies from pre-training to follow-up suggest that online delivery may hold promise in changing practitioners’ competencies and organizational practices. Encouragingly, practitioners also rated OT as very helpful at post-assessment.

This was the first study to examine training-related changes in organizational practices and support for father engagement. Both OT and FFT showed significant improvements from pre- to follow-up in the use of organizational strategies to engage fathers, and the FFT (but not OT) also showed improvements in organizational support for father-inclusive practices, in support of Hypothesis 3. There were only a few participants in the OT and FFT who were in management roles, so it appears unlikely that changes in organizational practices were a result of their participation in training and disseminating the strategies via a ‘top-down’ approach, which has been suggested as important in other father training research [6]. Instead it would appear that training practitioners to improve their skills can have ‘bottom-up’ effects on organizational practices to engage fathers, most likely because individuals share the information with their colleagues and are able to influence practices within the organization. Indeed, a specific focus of both the OT and FFT was for practitioners to identify ways of influencing their specific organization. Thus, this research suggests that training to enhance practitioner competencies in father engagement can also improve organizational practices, without necessarily requiring the involvement of managers to lead this change.

We found both programs to enhance competencies, organizational practices and practitioner reported father engagement; however, the mechanisms by which training practitioners to enhance their competencies results in changes in father engagement were not explored. Future research should attempt to examine this question, as identifying mechanisms of change may help to elucidate the critical components to include in training programs. Such research would be best conducted using an objective measure of father engagement (such as actual attendance rates), to avoid any biases in practitioner-rated reports of father engagement. It would also be important for future research to examine more distal outcomes of training, such as improvements in child and parenting outcomes as a consequence of greater rates of father engagement.

There are a number of implications of the present research for practice in relation to the provision of father engagement training. Firstly, on the basis of the existing low rates of father engagement and low levels of practitioner competencies [4], it seems important to provide training programs more widely to enhance practitioner and organizational father engagement practices. Previous research suggests that the majority of practitioners would participate in father engagement training if given the opportunity [4]. In both studies in the current research, less than one in five participants reported having received any previous training regarding engaging fathers. There may be an opportunity to include such training within University level courses, as highlighted by Fletcher and colleagues [27], so that father-inclusive practice is promoted from the very beginning of skill development. Secondly, the findings suggest that it is possible to develop and disseminate training programs to build skills across a broad range of professionals delivering parenting interventions, and discipline-specific training programs may not be needed. However, for practitioners working with some populations of fathers (e.g., fathers in the child protection system or culturally and linguistically diverse families) there may still be a need for specifically developed training programs which focuses on specific skill sets relevant to these populations. Thirdly, and relatedly, given that the positive effects of training appeared to generalize to organizational practices, there does not appear to be a need for separate training for managers in order to improve the organizational context as other researchers have advised [6], although once again the current finding may relate only to delivery of parenting interventions. Finally, online training formats have a number of benefits over face-to-face formats, in terms of reduced demands on practitioner time, ease of access and participation, ease of adaptation to specific groups of fathers, and significantly lower cost of delivery, and therefore may be a promising alternative to face-to-face programs, although further research is needed to identify strategies to enhance maintenance of training effects over time.

This research had a number of key strengths including large sample sizes in the two studies, use of follow-up assessment, and participation by diverse practitioners working in a wide range of organizations, as well as participants from all states and territories in Australia. However, there were a number of limitations. First, as the sample of practitioners were self-selected they may not be representative of practitioners delivering parenting interventions in the community, and therefore they may have had more positive attitudes to father engagement and thus demonstrated better outcomes. Second, there were relatively high rates of participant drop out, which may have positively influenced study findings, especially for the online training, as those who dropped out at follow-up had lower levels of pre-training confidence than those who remained. Third, the measure of father engagement was rated by practitioners, which may have introduced bias. Future research should endeavor to include objective measures of father engagement, as Scourfield et al. [6] did previously with the inclusion of data from case records. Few services keep routine records on father engagement [24], which highlights the importance of organizations routinely collecting data on rates of father engagement, so that the impact of training can be readily examined.

Finally, no research on father engagement training for practitioners has yet been conducted as part of a randomized controlled trial (RCT), and the inclusion of a control group is critical to ensure that confounds are controlled for. Relatedly, the present research included two separate studies, and a stronger design would have been to directly compare both training formats within the same study. However, due to differences between the two studies in samples (as a result of self-selection effects) and procedures (e.g., recruitment duration, length of time to post-training assessment), it was deemed not feasible to directly compare the effects of these training programs. Future research should aim to use RCTs, include objective measures of father engagement, and examine the mechanisms of change in training. Future studies should also aim to use follow-up periods beyond 2 months to examine maintenance of training effects in the longer-term. Future research on online training could explore strategies to support the implementation of skills following training and evaluate these, in an effort to maintain improvements in practitioner competencies over time.

## Summary

Overall, the results from the present research suggest that a brief online and face-to-face training program were both promising in improving father engagement practices and organizational practices. Thus, training for practitioners may represent a viable strategy for increasing the engagement of fathers in evidence-based parenting interventions. Given maintenance of training effects for face-to-face but not online training, the more intensive face-to-face version may confer stronger long-term outcomes than the online format. However, given the large effect sizes in improvements for online training, along with low cost and ease of participation, the online format has considerable potential to enhance rates of competencies, particularly for practitioners who cannot attend training in person. Further research on enhancing practitioner competencies to better engage fathers is imperative, especially given the low rates of father engagement in evidence-based parenting interventions.
